# Cognitive Groove Expands Life Space in Older Adults with Mild Cognitive Impairment: A Community‐Based Rehabilitation Approach

**DOI:** 10.1002/alz70861_108459

**Published:** 2025-12-23

**Authors:** George Ioannidis, Alexandra Papaioannou, Courtney Kennedy, Justin Y Lee, Sharon Marr, Genevieve Hladysh, Ashlee M Azizudin, Caroline Marr, Patricia Hewston

**Affiliations:** ^1^ McMaster University, Hamilton, ON Canada; ^2^ Geras Centre for Aging Research, Hamilton, ON Canada; ^3^ YMCA of Hamilton/Burlington/Brantford, Hamilton, ON Canada

## Abstract

**Background:**

Life space mobility ‐ how far and how often someone moves through their environment ‐ is a meaningful marker of physical function, cognitive health, and independence. In older adults with mild cognitive impairment (MCI), life space often becomes restricted and can accelerate functional decline. This study evaluated the impact of Cognitive Groove, brought to you by GERAS DANCE, on life space mobility in older adults with and without MCI.

**Method:**

Cognitive Groove is a structured, community‐based rehabilitation program designed for older adults with early cognitive and/or mobility challenges. The program was delivered across 12 YMCA sites in Southern Ontario, Canada. The program included in‐person dance classes (twice weekly, 1 hour each) and at‐home balance exercises (10 minutes daily) over 12‐weeks. Life space mobility was assessed using the Life Space Assessment (LSA), cognitive function with the Montreal Cognitive Assessment (MoCA), and physical function with the Short Physical Performance Battery (SPPB). MCI was defined as a baseline MoCA score of 18‐25. Paired t‐tests examined the changes from baseline to 12 weeks, and the chi‐square test assessed changes in LSA frequency. Statistical significance was *p* ≤0.05.

**Result:**

A total of 106 older adults (age = 76.10(7.0) years; range 61–93) participated in Cognitive Groove. There were 61 older adults classified with MCI and 45 without MCI. Program adherence was high, with an average attendance of 75% (18/24 sessions). By the end of the program, older adults with MCI were moving farther and more often in their daily lives (*p* ≤0.05). Their life space became similar to those without MCI, whose movement stayed the same during the program (*p* >0.05). Notably, older adults with MCI reported more frequent travel outside their neighbourhood but within their town (Life Space Level 4, *p* <0.001). MoCA scores improved by 1.04 points in the MCI group, exceeding the minimal clinically important difference of 1.0, and both groups demonstrated significant improvements in SPPB scores (*p* <0.001).

**Conclusion:**

These findings suggest that Cognitive Groove not only enhances physical performance, but also life space mobility, by fostering community re‐engagement among older adults with MCI to support functional independence and active aging.

